# Hepatic transcriptome analysis from HFD-fed mice defines a long noncoding RNA regulating cellular cholesterol levels[S]

**DOI:** 10.1194/jlr.M086215

**Published:** 2018-11-30

**Authors:** Qian Chen, Chaoliang Xiong, Kunyun Jia, Jing Jin, Ziyang Li, Yazhou Huang, Yewen Liu, Lingling Wang, Haitao Luo, Haiyan Li, Qing H. Meng, Wei Li

**Affiliations:** Key Laboratory of Laboratory Medicine, Ministry of Education of China, School of Laboratory Medicine and Life Science,* Wenzhou Medical University, Wenzhou 325035, China; Zhejiang Provincial Key Laboratory of Medical Genetics,†† Wenzhou Medical University, Wenzhou 325035, China; Key Laboratory of Intelligent Information Processing,† Advanced Computer Research Center, State Key Laboratory of Computer Architecture, Institute of Computing Technology, Chinese Academy of Sciences, Beijing 100190, China; Department of Rehabilitation Medicine,§ First Affiliated Hospital of Wenzhou Medical University, Wenzhou 325035, China; Department of Laboratory Medicine,** University of Texas MD Anderson Cancer Center, Houston, TX 77030

**Keywords:** gene expression, peroxisome proliferator-activated receptors, cholesterol/biosynthesis, insulin resistance, noncoding ribonucleic acids, ribonucleic acid sequencing

## Abstract

To elucidate the transcriptomic changes of long noncoding RNAs (lncRNAs) in high-fat diet (HFD)-fed mice, we defined their hepatic transcriptome by RNA sequencing. Aberrant expression of 37 representative lncRNAs and 254 protein-coding RNAs was observed in the livers of HFD-fed mice with insulin resistance compared with the livers from control mice. Of these, 24 lncRNAs and 179 protein-coding RNAs were upregulated, whereas 13 lncRNAs and 75 protein-coding RNAs were downregulated. Functional analyses showed that the aberrantly expressed protein-coding RNAs were enriched in various lipid metabolic processes and in the insulin signaling pathway. Genomic juxtaposition and coexpression patterns identified six pairs of aberrantly expressed lncRNAs and protein-coding genes, consisting of five lncRNAs and five protein-coding genes. Four of these protein-coding genes are targeted genes upregulated by PPARα. As expected, the corresponding lncRNAs were significantly elevated in AML12 cells treated with palmitic acid or the PPARα agonist, WY14643. In Hepa1-6 cells, knockdown of NONMMUG027912 increased the cellular cholesterol level, the expression of cholesterol biosynthesis genes and proteins, and the HMG-CoA reductase activity. This genome-wide profiling of lncRNAs in HFD-fed mice reveals one lncRNA, NONMMUG027912, which is potentially regulated by PPARα and is implicated in the process of cholesterol biosynthesis.

Long noncoding RNAs (lncRNAs) are defined as non-protein-coding transcripts greater than 200 nt in length. It has been estimated that at least four-fifths of transcription across the human genome is associated with long noncoding genes (1). Most of the lncRNAs are similar to mRNA molecules in features, including 5′ capping, splicing, and polyadenylation. However, lncRNA sequences are generally less conserved across species than coding transcripts (2). lncRNAs are transcribed either from the intergenic genomic region independently or as complex interlaced networks of overlapping sense and antisense transcripts from protein-coding genes. Furthermore, lncRNA gene expression is often tissue-specific and varies among tissues to a greater degree than protein-coding genes, suggesting that lncRNA genes might carry out specific functions (3).

lncRNAs have been implicated in diverse processes of gene regulation, such as chromatin modification, direct transcription regulation, RNA processing, posttranslational regulation of protein activity, and localization as well as modulation of microRNA action (4). There is increasing evidence that lncRNAs are involved in various essential physiological functions and pathological processes, including X chromosome inactivation, genomic imprinting, adipogenesis, tumorigenesis, and cardiovascular disorders (5). However, the function and activity of the majority of lncRNAs remain poorly understood, even though we are now able to detect their presence.

The liver plays a crucial role in the integration of diverse metabolic processes, including glucose and lipid metabolism, bile acid synthesis, detoxification of xenobiotic compounds, and the secretion of plasma proteins (6). Hepatic metabolism is highly regulated by nutritional and hormonal factors to maintain nutrient and energy homeostasis in the body. Extensive metabolic changes can be induced in the liver by a high-fat diet (HFD), resulting in nonalcoholic fatty liver disease (NAFLD), insulin resistance, dyslipidemia, and obesity (7). With the rapidly growing epidemic of metabolic syndrome globally, discovery of effective interventions is essential. Numerous efforts are currently underway to improve our understanding of the molecular networks involved in cellular glucose, lipid, and energy metabolism in the liver.

Emerging evidence suggests that lncRNAs regulate hepatic lipid metabolism via various pathways. A liver-enriched lncRNA, lncLSTR, forms a complex with TAR DNA-binding protein 43 (TDP-43) to regulate the expression of sterol 12α-hydroxylase (Cyp8b1), inducing Apoc2 expression through a FXR-mediated pathway. Depletion of lncLSTR reduced triglyceride levels in a mouse model of hyperlipidemia (8). Another lncRNA, HULC, increased the expression of PPARα, which activated acy1-CoA synthetase long-chain family member 1 (ACSL1) and suppressed miR-9 by inducing the methylation of the CpG island at its promoter. This stimulated the accumulation of intracellular triglycerides and cholesterol in vitro and in vivo (9). A third lncRNA, LeXis, interacts with RALY, a transcription cofactor for genes involved in cholesterol biosynthesis, to affect the expression of these genes and change the cholesterol levels in mouse livers and plasma (10). Moreover, an imprinted lncRNA, MEG3, serves as a guide RNA scaffold to recruit polypyrimidine tract-binding protein 1 (PTBP1) to destabilize Shp mRNA, inducing cholestatic liver injury (11).

In this study, we investigated the potential role of lncRNAs in lipid metabolism. As RNA sequencing (RNA-seq) has been generally used for transcriptome construction, it was used in our study to interpret the global features of all RNA transcripts, both protein-coding and noncoding RNAs. A mouse model was established by feeding mice a HFD, and the transcriptome was determined by RNA-seq and systematic analysis. Further, one candidate lncRNA was subjected to cell assays.

## MATERIALS AND METHODS

### Animals

Male C57BL/6J mice (n = 24) were obtained from the Shanghai Laboratory Animal Center at 12 weeks of age and housed in the Experimental Animal Center of Wenzhou Medical University. All animal experiments were conducted in accordance with the recommendations in the *Guide for the Care and Use of Laboratory Animals* of the National Institutes of Health and was in line with the global 3R (reduce, reuse, and recycle) Initiative. All protocols were approved by the Committee on the Ethics of Animal Experiments of Wenzhou Medical University.

The mice were acclimatized to the housing conditions for 1 week, and then they were randomly assigned into two groups that were matched for age and weight. One group was maintained on a chow diet as the control, and the other was fed a HFD (MD12032; Medicience Ltd., Jiangsu, China). The composition of the diet is described in supplemental Table S1.

Both groups were fed for 3 months and body weight was measured monthly. After 3 months, the mice were subjected to glucose tolerance and insulin sensitivity tests and then were euthanized. The liver, heart, spleen, lung, kidney, muscle, intestine, fat, and brain were harvested and frozen in liquid nitrogen for further analysis.

### Measurement of total hepatic cholesterol

Liver tissue was weighed and added to homogenization medium (absolute ethyl alcohol for the HFD group) in a mass-to-volume ratio of 1:9 (g/ml). Following mechanical homogenization in an ice bath, the samples were centrifuged at 600 *g* for 10 min, and the supernatants were added to 96-well plates using a T-CHO testing kit (Jiancheng Bioengineering Institute, Nanjing, China). The plate was then incubated at 37°C for 3 min and the absorbance at 510 nm was detected using a microplate spectrophotometer. The protein concentration was detected with the BCA method.

### Cell culture and transfection

Cell lines (AML12 and Hepa1-6) were obtained from the Stem Cell Bank, Chinese Academy of Sciences (Shanghai, China). AML12 cells were cultured in DMEM/F-12 medium (Gibco; #11320033) with 10% (v/v) FBS (Gibco; #10100147), 1% (v/v) ITS liquid media supplement (Sigma; #I3146-5ML), and 40 ng/ml dexamethasone (Sigma; #D4902-25MG). In addition, AML12 cells were treated with 700 μM palmitic acid (PA) (Sigma; #P0500-10G) following culture in 2% (v/v) fatty acid-free BSA (Sigma; #A6003-25G) for 16 h, and in WY14643 (Selleck; #S8029) with a final concentration of 300 μM in DMEM/F-12 for 12 h.

Hepa1-6 cells were cultured in DMEM with 10% (v/v) FBS. Hepa1-6 cells were transfected with 10 μM siRNA (RiboBio, Guangzhou, China) or scrambled siRNA (Life Technologies) with Lipofectamine 2000 according to the manufacturer’s protocol (Lipofectamine™ RNAiMAX; Invitrogen). The negative control siRNA has no significant sequence similarity to mouse, rat, or human gene sequences. The control had been tested previously in cell-based screens and proven to have no significant effect on cell proliferation, viability, or morphology. This reagent was of a high purity, as is required for animal use.

After 48 h of transfection, the cellular cholesterol level was measured using a kit (Jiancheng Bioengineering Institute, Nanjing, China). Cellular lipid droplets were detected by Oil Red O staining as previously described (12).

### Cell viability

AML12 cells were seeded into 96-well plates at a density of 5,000 cells/well (100 μl total volume) and incubated at 37°C and 5% CO_2_ for 24 h. Cells were treated with different concentrations of PA (300 μM, 700 μM, and 800 μM). After 16 h, 10 μl of 5 mg/ml methyl thiazolyl tetrazolium were added to each well and incubated for 4 h. The medium was then removed and the precipitated formazan was dissolved in 100 μl of DMSO followed by shaking for 10 min. Finally, the absorbance at 570 nm was measured on a microplate spectrophotometer.

### RNA extraction, library construction, and high-throughput sequencing

Total RNA was isolated using the TRIzol reagent (Invitrogen; #15596026), treated with DNase I, and purified using RNeasy Mini columns (Qiagen; #74034). The purity and quantity of the total RNA was measured using a Nanodrop 2000 (Thermo Scientific). The integrity of the RNA was assessed using a Bioanalyzer 2000 system (Agilent Technologies).

The library was constructed according to Novogene’s (Beijing, China) procedure, as previously described (13). Briefly, 3 μg of total RNA were utilized for the RNA sample preparations. rRNA was removed using the Ribo-zero™ rRNA removal kit (Epicentre) and the residual RNAs were cleaned up by ethanol precipitation. Subsequently, sequencing libraries were generated based on the rRNA-depleted RNA by using NEBNext® Ultra™ directional RNA library prep kit from Illumina® (Lincoln, NE) following the manufacturer’s recommendations. After library quality was assessed on an Agilent Bioanalyzer 2100 system, RNA-seq was performed on the Illumina Hiseq 2500 platform by Novogene.

### Read mapping and transcriptome assembly

Raw reads were processed by removing the adaptor reads or ploy-N and low-quality reads to obtain the high-quality clean reads. Q20, Q30, and GC content of the clean data were subsequently calculated. All clean reads were mapped to the mouse reference genome (mm9) with TopHat software (version 2.0.6; http://tophat.cbcb.umd.edu/) using the default parameters. In detail, all reads that mapped directly to the genome (with no gaps) were aligned using Bowtie software (http://bowtie-bio.sourceforge.net/index.shtm/). Then, the remaining reads that were not aligned were mapped using a gapped alignment algorithm. Only uniquely mapped and properly paired reads were subjected to Cufflinks software (version 2.0.2; http://cufflinks.cbcb.umd.edu/) for ab initio transcript assembly. To produce a unique set of transcriptomes, the multiple assembled transcripts from each sample were merged using the Cuffmerge utility provided in the Cufflinks package.

### Identification of lncRNAs

lncRNAs were identified using an established procedure developed by Luo et al. (14). The method implements a highly stringent step-by-step filtering procedure to minimize false positives and maximize the detection of bona fide lncRNAs. The pipeline is briefly summarized as follows: *1*) Transcript comparison: merged transcripts were compared with known gene annotations (i.e., RefSeq for coding genes, NONCODE for noncoding genes), relying on the Cuffcompare utility included in the Cufflinks suite. *2*) Classification of coding and noncoding transcripts: for all potential novel transcripts, a CNCI score was calculated with CNCI software (15), which has been proven to be the most effective lncRNA identification software. *3*) Filter novel lncRNA genes: putative lncRNA genes were identified by considering the length of transcripts (length ≥200 nt), CNCI score (score <0), and exon number (exon number ≥2).

### GO and KEGG enrichment analysis of differentially expressed genes

Cuffdiff (a module in the Cufflinks package) was employed to identify the differentially expressed genes (DEGs) with thresholds of fold-change >2.0 or <0.5 and a *P* value of <0.05. Hierarchical clustering was performed to display the distinguishable expression patterns of the mRNAs and lncRNAs among samples.

Gene symbols of the differentially expressed coding genes were input into DAVID (http://david.abcc.ncifcrf.gov/) for gene ontology (GO) and Kyoto Encyclopedia of Genes and Genomes (KEGG) annotation (16). For the lncRNA functions, the coexpression analysis between the differentially expressed lncRNAs and genomic juxtaposition protein-coding RNAs was performed to predict the potential functions for lncRNAs as previously reported (17). A Pearson correlation coefficient >0.90 with a *P* value of <0.05 indicated a significant relationship. Furthermore, we used the RPISeq web server (http://pridb.gdcb.iastate.edu/RPISeq/index.html) to verify this regulatory relationship (18).

### Subcellular localization of lncRNA NONMMUG027912

Fluorescence in situ hybridization (FISH) analysis was performed using a fluorescent in situ hybridization kit (RiboBio, Guangzhou). The FISH probes for lncRNA NONMMUG027912 were designed and synthesized by RiboBio. Probes to the 18S rRNA and U6 were provided as the cytoplasmic- and nuclear-positive controls, respectively. All probes were labeled with Cy3 and hybridization was performed according to the manufacturer’s instructions, as previously reported (19). After hybridization, the imaging was done immediately after DAPI staining using a laser scanning confocal microscope (Nikon, Japan).

Nuclear and cytoplasmic RNA separation and purification were accomplished with an RNA purification kit (Norgen Biotek, Canada) according to the manufacturer’s instructions. After reverse transcription, NONMMUG027912 was detected by quantitative real-time PCR and the relative ratios of nuclear and cytoplasmic RNA were determined via a method described previously (20).

### Histology examination

For H&E staining, the slides were first incubated with hematoxylin and then washed with 1% (v/v) ethanol hydrochloride. After washing with water, the slides were stained with eosin and dehydrated with an alcohol gradient. The H&E sections were examined by a pathologist according to the NAFLD activity score, which is defined as the unweighted sum of the scores for steatosis (0–3), lobular inflammation (0–3), and ballooning (0–2), with a score of ≥5 corresponding to steatohepatitis (21).

### HMG-CoA reductase activity assay

HMG-CoA reductase activity was detected using the HMG-CoA direct spectrophotometry quantitative detection kit (HL50137.1; Shanghai Haling Biotechnology Co. Ltd.). Briefly, after 48 h of siRNA transfection, the culture medium of 1–5 × 10^6^ Hepa1-6 cells was removed and 1 ml of reagent A (cleaning buffer) was added to cover the growth surface. Subsequently, reagent A was removed and the cells were detached from culture dishes using CellStripper (Fisher Scientific). Then, 1 ml of reagent A was added to the cells and transferred to a precooled conical centrifuge tube, where it was then spun at 300 *g* for 5 min at 4°C. The supernatant was removed and 500 μl of reagent B (lysis buffer) were added to the precipitate. The solution was transferred to a 1.5 ml Eppendorf tube and vortexed for 15 s. The sample was then centrifuged at 16,000 *g* for 10 min at 4°C. The supernatant (500 μl) was added to the protein enrichment tube and centrifuged at 7,500 *g* for 35 min at 4°C. The protein concentration was determined via the BCA method.

### Quantitative RT-PCR

Total RNA was isolated from tissues or cells using the TRIzol reagent (Invitrogen; #15596026). After treatment with recombinant DNase I (RNase-free) (Takara; 2270A), cDNA was synthesized using the SuperScript III First-Strand synthesis system (Invitrogen; #18080051). Quantitative RT-PCR was performed on a CFX96TM real-time PCR system (Applied Biosystems). The PCR reagent mixture was obtained from Invitrogen (#11744500). The PCR program used was as follows: 10 min at 95°C, 40 cycles of 15 s at 95°C, and 1 min at 60°C. Melting curve analysis was performed to confirm the real-time PCR products. The primer sequences used are listed in supplemental Table S2.

### Western blot analysis

Cells were collected 48 h posttransfection and lysed with cell lysis buffer for 30 min. The lysate protein concentration was measured using a Bradford protein assay kit (Bio-Rad) and the proteins were then separated via sodium dodecyl sulfate polyacrylamide gel electrophoresis in 12% sodium dodecyl sulfate gels and then transferred to PVDF membranes (Millipore, Billerica, MA). Immunoblotting was performed using select primary antibodies, followed by probing with the recommended secondary antibodies. β-Actin was used as a control. Finally, the signals were detected using an Odyssey infrared imaging system (LI-COR, Lincoln, NE).

### Statistical analysis

All experiments were repeated three times, and the results were presented as mean ± SD. Statistical analyses were performed using GraphPad Prism 7.0 (GraphPad Software, San Diego, CA). The *P* values were calculated using a one-way ANOVA. A *P* value of <0.05 was considered statistically significant.

## RESULTS

### HFD-induced nonalcoholic steatohepatitis with insulin resistance in mice

**Fig. 1.**
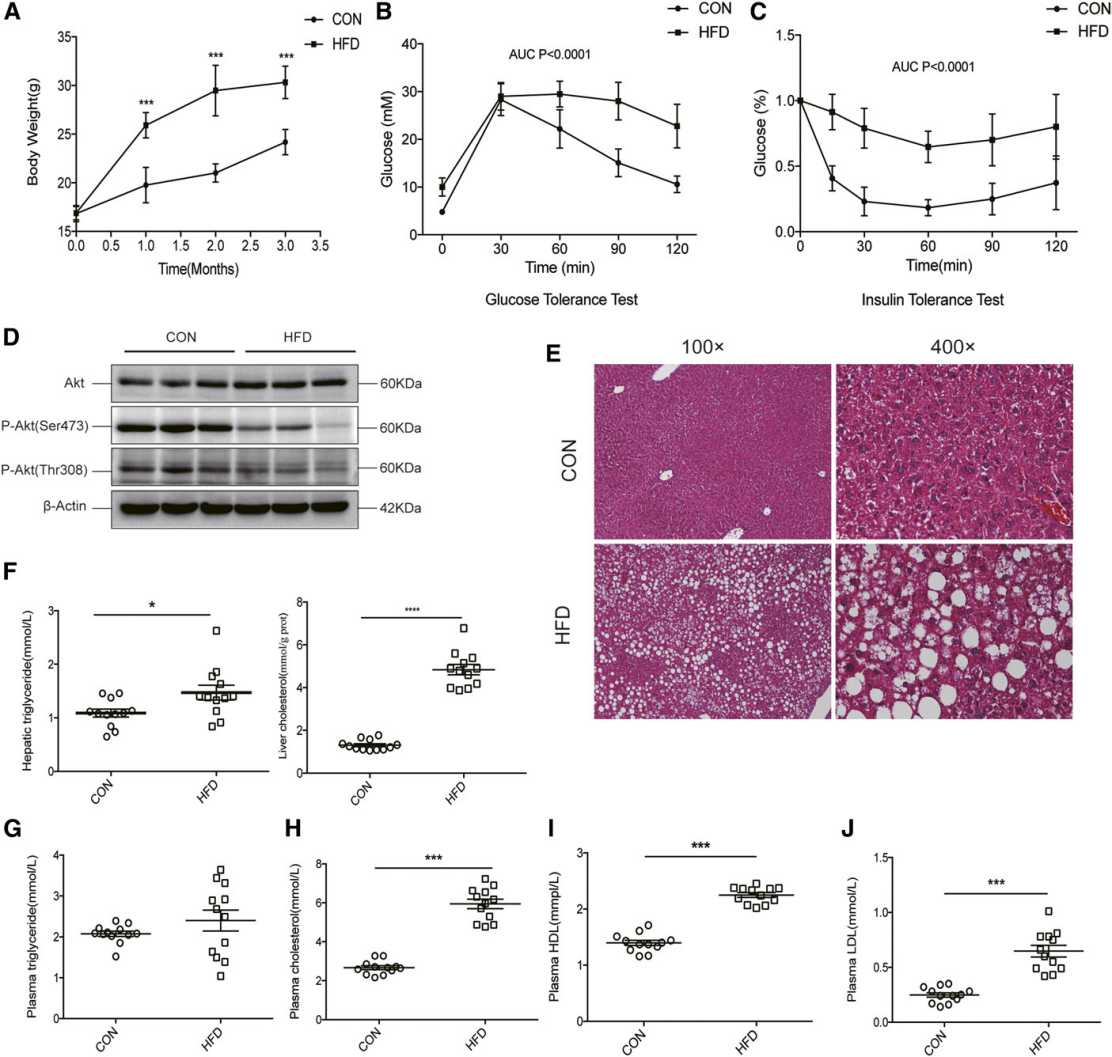
Nonalcoholic steatohepatitis with insulin resistance induced by a HFD. A: The body weights of mice fed a HFD were significantly increased as compared with control mice after 3 months (males, n = 12, ***P < 0.001). B, C: AUC measurements of glucose tolerance and insulin sensitivity tests show an impaired insulin sensitivity in mice fed a HFD as compared with controls (*P* < 0.001). D: Phosphorylation of Akt (Ser473 and Thr308) was significantly decreased in livers from mice fed a HFD when compared with control mice (n = 3). E: HFD mice showed remarkable liver fat accumulation as observed by H&E staining (n = 3). F: Liver triglyceride and cholesterol levels were elevated in the HFD mice as compared with control mice (n = 12, *P < 0.05). G: There was no significant difference in the plasma triglyceride levels between HFD and control mice (*P* > 0.05). Total cholesterol (H), HDL (I), and LDL (J) levels were elevated in HFD mice as compared with controls (n = 12, ***P < 0.001).

After 3 months, the body weights of HFD-fed mice were significantly higher than those of chow-fed control mice (**Fig. 1A**). Additionally, the HFD-fed mice showed markedly lower glucose tolerance and insulin sensitivity (Fig. 1B, C). Moreover, the phosphorylation of Akt at the T308 and S473 sites in liver tissues was remarkably decreased in the HFD-fed mice after insulin stimulation, which indicated that the insulin signaling pathway was impaired (Fig. 1D). Notably, H&E staining of liver tissues showed that HFD-fed mice had steatohepatitis (Fig. 1E, supplemental Table S3), which is supported by the NAFLD activity score (21). Moreover, triglyceride and cholesterol content in the liver was significantly increased in the HFD mice (Fig. 1F), and plasma biochemical indices indicated the emergence of dyslipidemia in these mice, with increased levels of plasma triglycerides, cholesterol, HDLs, and LDLs (Fig. 1G–J).

### Hepatic transcriptome atlas of HFD-induced nonalcoholic steatohepatitis in mice

**Fig. 2.**
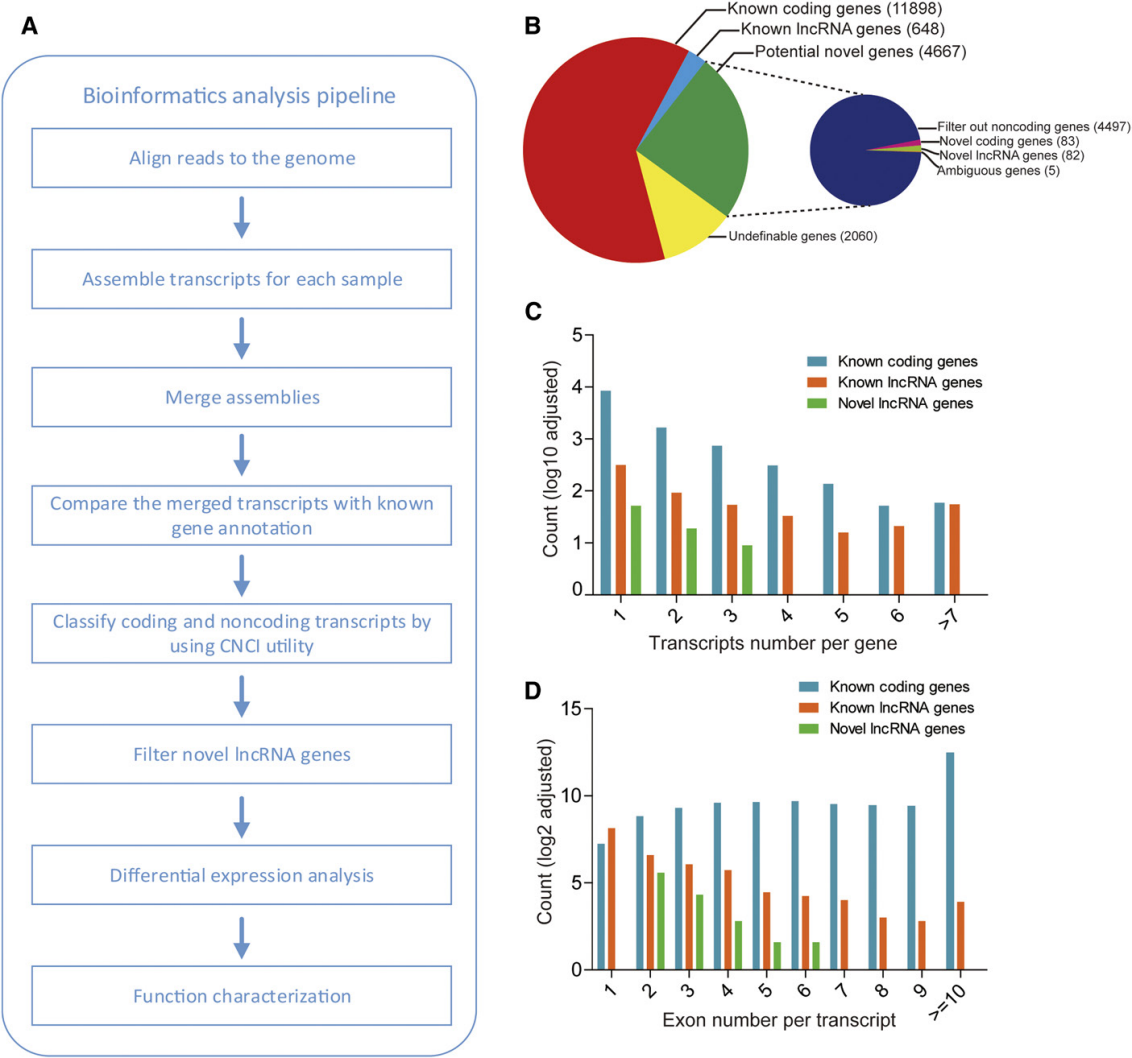
Bioinformatics analysis pipeline and classification of identified transcripts. A: The pipeline employed in the identification and function annotation of protein-coding and noncoding RNAs. B: Classification and statistics of protein-coding and noncoding genes identified using the CNCI utility (potential novel genes were additionally annotated in the right smaller pie). C: Distribution by transcript number per gene for identified known genes and novel lncRNA genes. D: Distribution by exon number per transcript for identified known genes and novel lncRNA genes.

To obtain a comprehensive transcriptome atlas of the fatty liver in the HFD mice, RNA-seq was carried out (**Fig. 2A**). In total, 326,645,424 paired-end reads with lengths of 125 base pairs were obtained after filtering out low-quality reads and removing the adaptor sequences. Mapping of the obtained clean reads to the mouse reference genome revealed that approximately 85% of the reads were aligned onto the mouse genome (mm9) (supplemental Table S4). The ab initio assembly transcripts derived from these reads numbered about 25,000 per sample; these transcripts were merged to constitute the more integrated and unified assembly totaling 33,803 transcripts (aligned to 19,274 genes) (supplemental Table S5). The total transcripts were aligned to 19,274 genes, of which 12,546 corresponded to known genes, 4,667 were novel, and the other 2,060 corresponded to undefinable genes (Fig. 2B).

The CNCI utility was employed to identify novel lncRNA genes, including considerations of their coding potential, exon number, and transcript length using multiple stringent criteria (15). Ultimately, the 1,247 lncRNA transcripts were associated with a total of 730 lncRNA genes, including 648 known and 82 novel lncRNAs (Fig. 2B). Among these 730 lncRNAs, 183 had alternative splicing, whereas 547 contained a unique transcript isoform. Some of these lncRNA genes contained a canonical splice site (GT-AG). Moreover, the amount of alternative splicing for lncRNAs was significantly less than that for protein-coding genes (Fig. 2C), and the average number of exons for lncRNAs was also significantly lower than that for coding genes (Fig. 2D), which was consistent with a previous study (2).

### Overview of hepatic protein coding and lncRNA gene expression profiles in mice

**Fig. 3.**
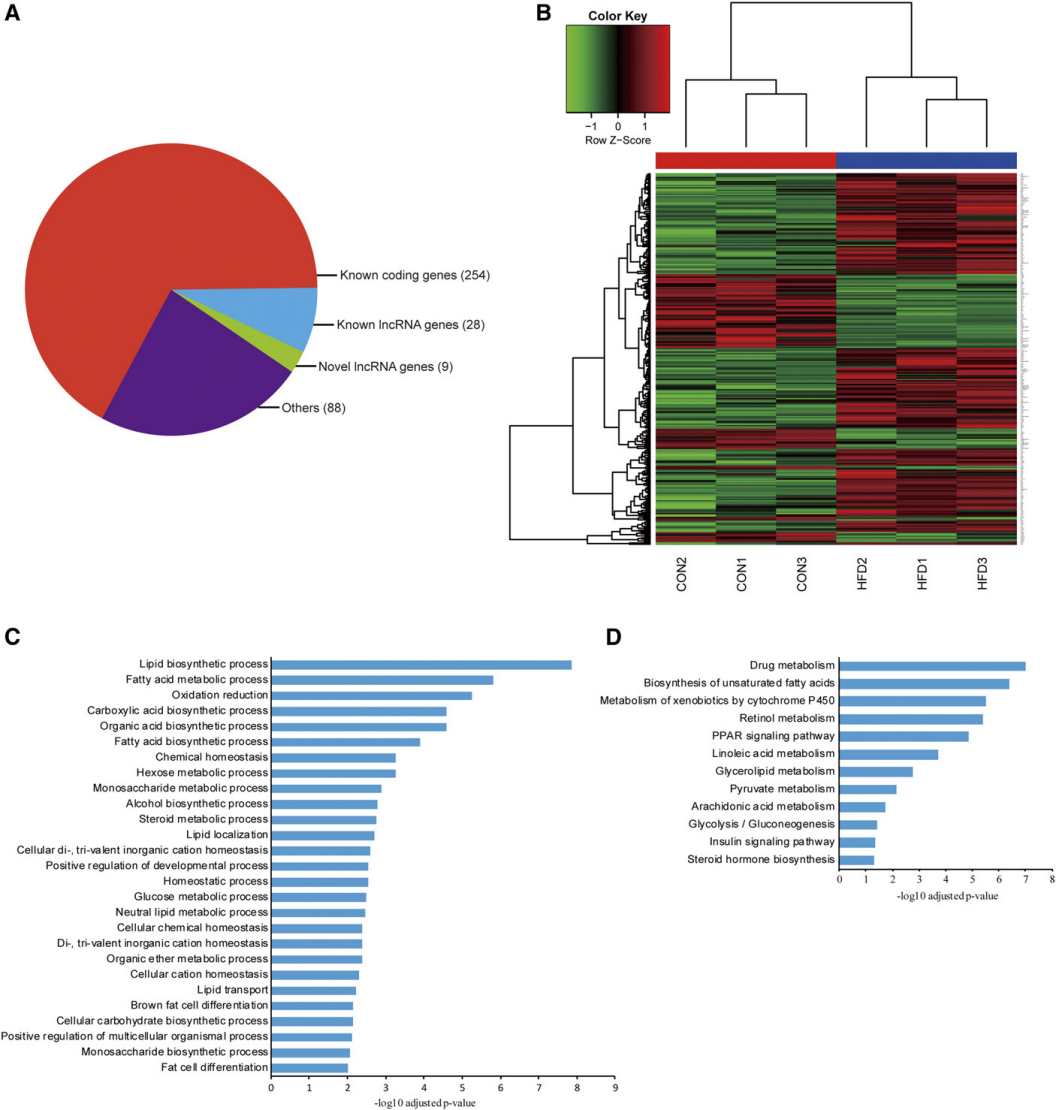
Classification and functional annotation of DEGs. A: Classification of DEGs in the livers of HFD and control mice. B: Cluster analysis for differentially expressed protein-coding genes. C: Enrichment in biological processes of differentially expressed protein-coding genes by GO analysis. D: KEGG pathway analysis of the differentially expressed protein-coding genes.

To obtain an overview of the expression profiles of both protein-coding genes and noncoding genes in these mouse livers, we used Cufflinks to quantify the expression levels of these genes across six liver samples from HFD-fed and control groups (n = 3 in each group). Compared with the livers from the control group, those from the HFD-fed mice featured 379 DEGs (**Fig. 3A**, B), including 254 known protein-coding genes (179 upregulated and 75 downregulated) and 37 lncRNAs (24 upregulated and 13 downregulated) (**Table 1**). GO and KEGG pathway analyses were performed to predict the function of the differentially expressed coding genes. GO analysis indicated that these genes were significantly overrepresented in 27 biological processes at *P* < 0.01, of which lipid biosynthetic processes, fatty acid metabolic processes, and oxidation reduction were the most significant (Fig. 3C). Additionally, differentially expressed coding genes were significantly enriched in 12 KEGG pathways, such as drug metabolism, biosynthesis of unsaturated fatty acids, and the metabolism of xenobiotics by cytochrome P450. Furthermore, the HFD-fed mouse model established here was confirmed at the transcriptional level by the enhancement of lipid synthesis and the reduction in the insulin pathway (Fig. 3D).

**TABLE 1.** Number of differentially expressed lncRNA and protein-coding genes in the livers of mice fed chow or a HFD

	Fold-Change ≥2	Fold-Change ≥4	Fold-Change ≥8
lncRNA genes			
Upregulated	24	14	2
Downregulated	13	8	4
Protein-coding genes			
Upregulated	179	39	12
Downregulated	75	18	5

### Functional characterization of differentially expressed lncRNAs

**Fig. 4.**
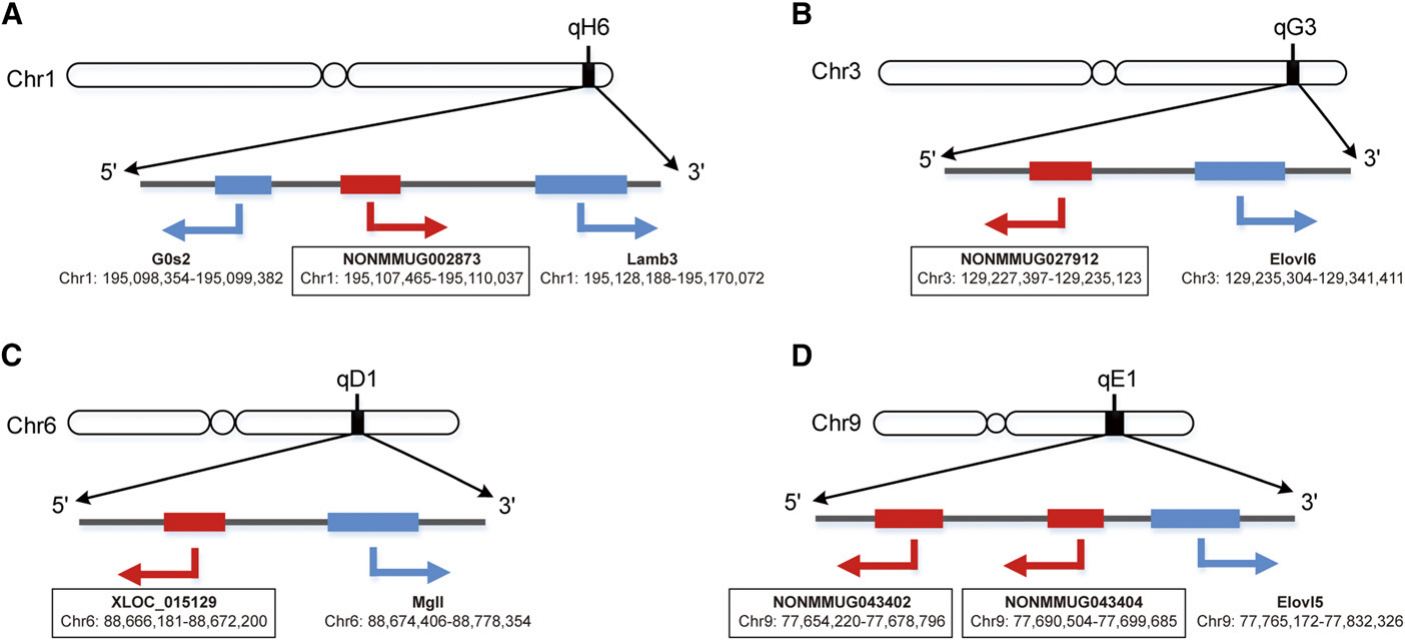
lncRNA and protein-coding gene pairs based on genomic juxtaposition and coexpression.

Because genes with similar coexpression patterns tend to exhibit functional coherency (22), and lncRNAs usually regulate their genomically juxtaposed genes, it may be feasible to infer the biological functions of lncRNAs based on their level of coexpression and orientation in the genome relative to coding genes (17, 23–25). For each of the 37 differentially expressed lncRNAs, we calculated a coexpression matrix between differentially expressed lncRNAs and differentially expressed protein-coding genes by computing the Spearman correlation across all samples. The lncRNA-protein-coding gene pairs with a Spearman correlation coefficient of *r* ≥ 0.8 and *P* < 0.05 were regarded as coexpressed, while those with a Spearman correlation coefficient of *r* ≥ 0.9 and *P* < 0.05 were considered to have strongly correlated expression. In total, we identified 3,045 coexpressed lncRNA-protein-coding gene pairs, 1,450 of which showed strong coexpression. For these coexpressed pairs, we further analyzed their genome orientation, assigning each pair within a 100 kb range of each other as *cis* functional pairs. As a result, six genomically juxtaposed and coexpressed lncRNA-protein-coding gene pairs were identified, comprising five lncRNAs and five protein-coding genes (**Table 2**, **Fig. 4**). Among the six pairs, three pairs (NONMMUG002873, *Lamb3*; NONMMUG027912, *Elovl6*; and NONMMUG043402, *Elovl5*) showed strong coexpression correlation (*r* ≥ 0.9), while the other three pairs (NONMMUG002873, *G0s2*; XLOC_015129, *Elovl6*; and NONMMUG043404, *Elovl5*) showed less strong coexpression correlation (*r* ≥ 0.8).

**TABLE 2.** lncRNA and protein-coding gene pairs based on genomic juxtaposition and coexpression

lncRNAs	Chromosome Location	Coding Genes	Chromosome Location	Distance (Kb)	Correlation Coefficient
NONMMUG002873	chr1:195107465-195110037	*G0s2*	chr1:195098354-195099382	8.0	0.77
NONMMUG002837	chr1:195107465-195110037	*Lamb3*	chr1:195128188-195170072	18.1	0.78
NONMMUG027912	chr3:129227397-129235123	*Elovl6*	chr3:129235304-129341411	0.18	0.91
XLOC_015129	chr6:88666181-88672200	*Mgll*	chr6:88674406-88778354	2.2	0.92
NONMMUG043402	chr9:77654220-77678796	*Elovl5*	chr9:77765172-77832326	86.3	0.95
NONMMUG043404	chr9:77690504-77699685	*Elovl5*	chr9:77765172-77832326	65.4	0.95

### Validation of the putative lncRNA-protein-coding gene pairs and prediction of the lncRNA function and activity

**Fig. 5.**
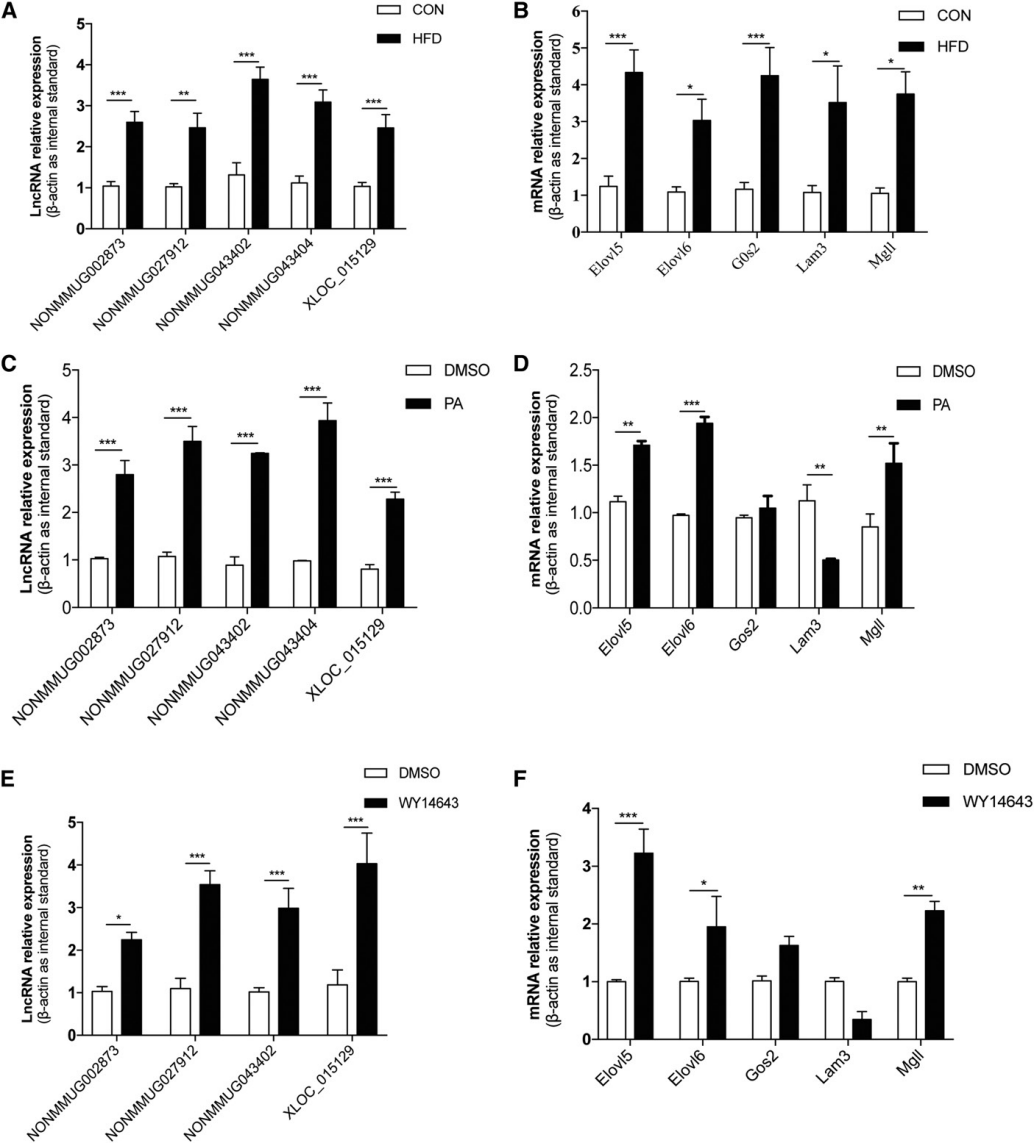
Expression of selected genes validated by qPCR and cellular assays. The upregulation of selected lncRNAs (A) and protein-coding genes (B) in HFD livers was validated by qPCR (n = 10; *P < 0.05, **P < 0.01, ***P < 0.001). Treatment of AML12 cells with PA (700 μM) for 16 h resulted in the upregulation of selected lncRNAs (C) and protein-coding genes *Elovl5*, *Elovl6*, and *Mgll* (n = 3, **P < 0.01, ***P < 0.001), as well as the downregulation of *Lamb3* (n = 3, **P < 0.01) (D). Treatment of AML12 cells with WY14643 (300 μM) for 12 h resulted in the upregulation of four selected lncRNAs (n = 3; *P < 0.05, ***P < 0.001) (E) and three protein-coding genes *Elovl5*, *Elovl6*, and *Mgll* (*P < 0.05, **P < 0.01, ***P < 0.001) (F).

The transcriptional levels of the five lncRNAs and five protein-coding genes identified in the coexpression analysis were detected by qPCR using total RNA extracted from the liver tissues of the HFD-fed and control mice. These experiments indicated that both the lncRNAs and the protein-coding RNAs were significantly upregulated in the HFD-fed mice as compared with the control mice (**Fig. 5A**, B), which was consistent with our RNA-seq result.

To mimic the in vivo process, we treated AML12 cells with the saturated fatty acid, PA. First, we investigated to determine whether the high concentration (700 μM) of PA was cytotoxic via the MTT assay. The results showed that, compared with the positive control group (10% DMSO), 700 μM PA was not toxic to the cells (supplemental Fig. S1). As shown in Fig. 5C, PA treatment significantly elevated the levels of the five lncRNAs. However, among the protein-coding RNAs, PA treatment remarkably increased the expression of *Elovl5*, *Elovl6*, and *Mgll*, but significantly decreased the level of *Lamb3*. Interestingly, PA had no effect on *G0s2* expression (Fig. 5D). The results indicate that the expression of the identified lncRNAs was affected by the saturated fatty acid, PA, and thus may be involved in fatty acid metabolism.

Interestingly, three of the five coding genes identified by the genomic juxtaposition and coexpression analysis, *Elovl5*, *Elovl6*, and *Mgll*, are target genes upregulated by PPARα in the mouse liver (supplemental Fig. S2) (26); Elovl5 and Elovl6 are involved in lipogenesis, and Mgll is expected to be involved in the breakdown of hepatic triglycerides to fatty acids (27). Thus, it was inferred that the five lncRNAs identified here might be regulated by PPARα due to their coexpression pattern. In AML12 cells treated with the PPARα agonist, WY14643, the predicted lncRNAs, NONMMUG002873, NONMMUG027912, NONMMUG043402, and XLOC_105129, as well as the protein-coding genes, *Elovl5*, *Elovl6*, and *Mgll*, were significantly elevated (Fig. 5E, F), as expected (28). These results indicated that NONMMUG002873, NONMMUG027912, NONMMUG043402, and XLOC_105129 were potentially regulated by PPARα, suggesting the possible roles for these lncRNAs in hepatic lipid metabolism.

### Characterization of NONMMUG027912 and its effects on lipid metabolism in Hepa1-6 cells

**Fig. 6.**
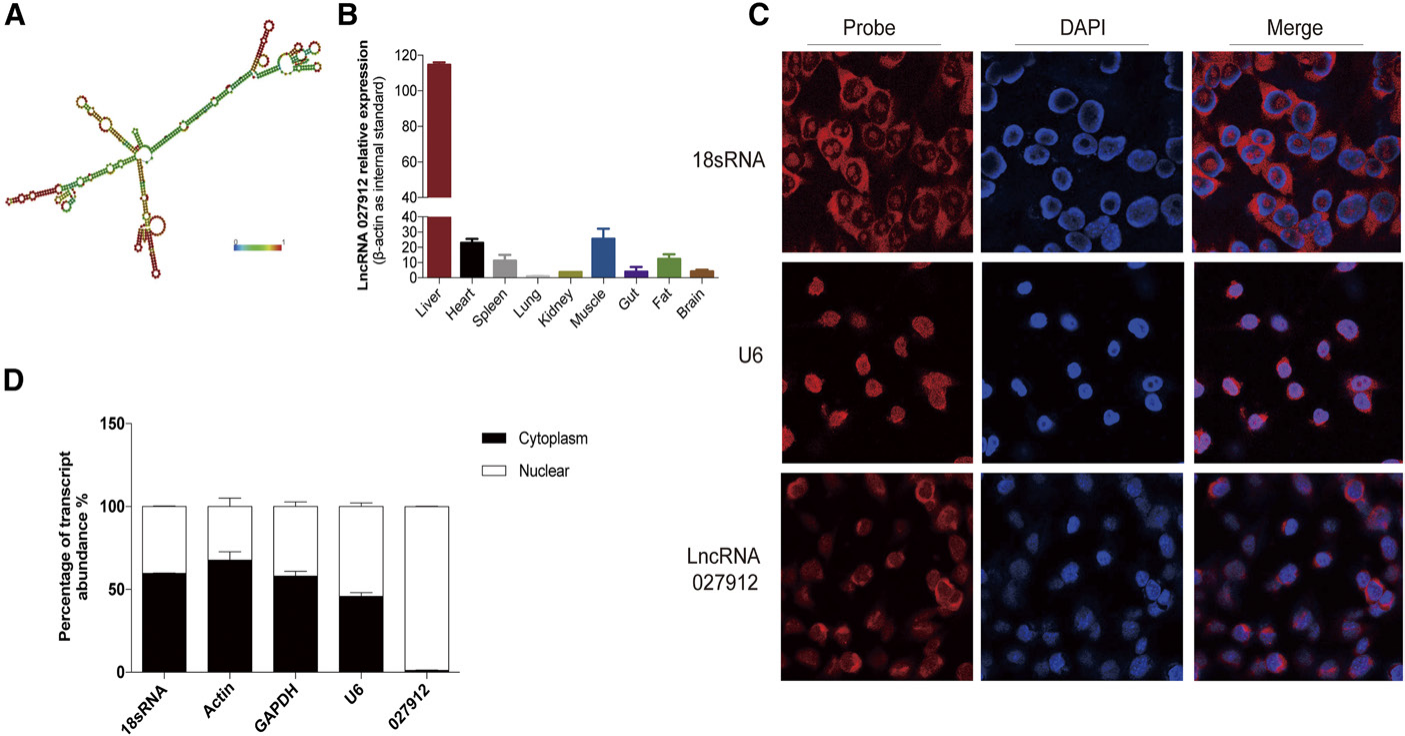
Characterization of NONMMUG027912. A: Prediction of the NONMMUG027912 secondary structure, which indicated the peculiar stem-loop structures identified on the RNAfold WebServer (http://rna.tbi.univie.ac.at/cgi-bin/RNAWebSuite/RNAfold.cgi). B: Tissue-specific expression of NONMMUG027912, as quantified by qPCR. The liver was the main site of NONMMUG027912 expression. C: FISH showed that NONMMUG027912 was localized predominantly in the nucleus of Hepa1-6 cells. D: Nuclear/cytoplasm RNA fractionation verified the nuclear localization of NONMMUG027912.

To confirm that the approach detailed above was effective in identifying lncRNAs that were potentially involved in hepatic lipid metabolism, one of these lncRNAs, NONMMUG027912, which is adjacent to *Elovl6* on mouse chromosome 3, was selected for the investigation of its effects on the lipid pathways in the liver. Its location on the chromosome was 129, 235, 304-129, 341, and 411 (Table 2). RNA secondary structure prediction showed that it had many peculiar stem-loop structures, suggesting the existence of DNA-, RNA-, or protein-binding sites (**Fig. 6A**).

We detected the expression of NONMMUG027912 in nine tissues, including liver, heart, spleen, lung, kidney, muscle, intestine, fat, and brain. As shown in Fig. 6B, it had the highest expression level in the liver. Both FISH and nuclear/cytosol fractionation (Fig. 6C, D) indicated that NONMMUG027912 was predominantly located in the nucleus.

**Fig. 7.**
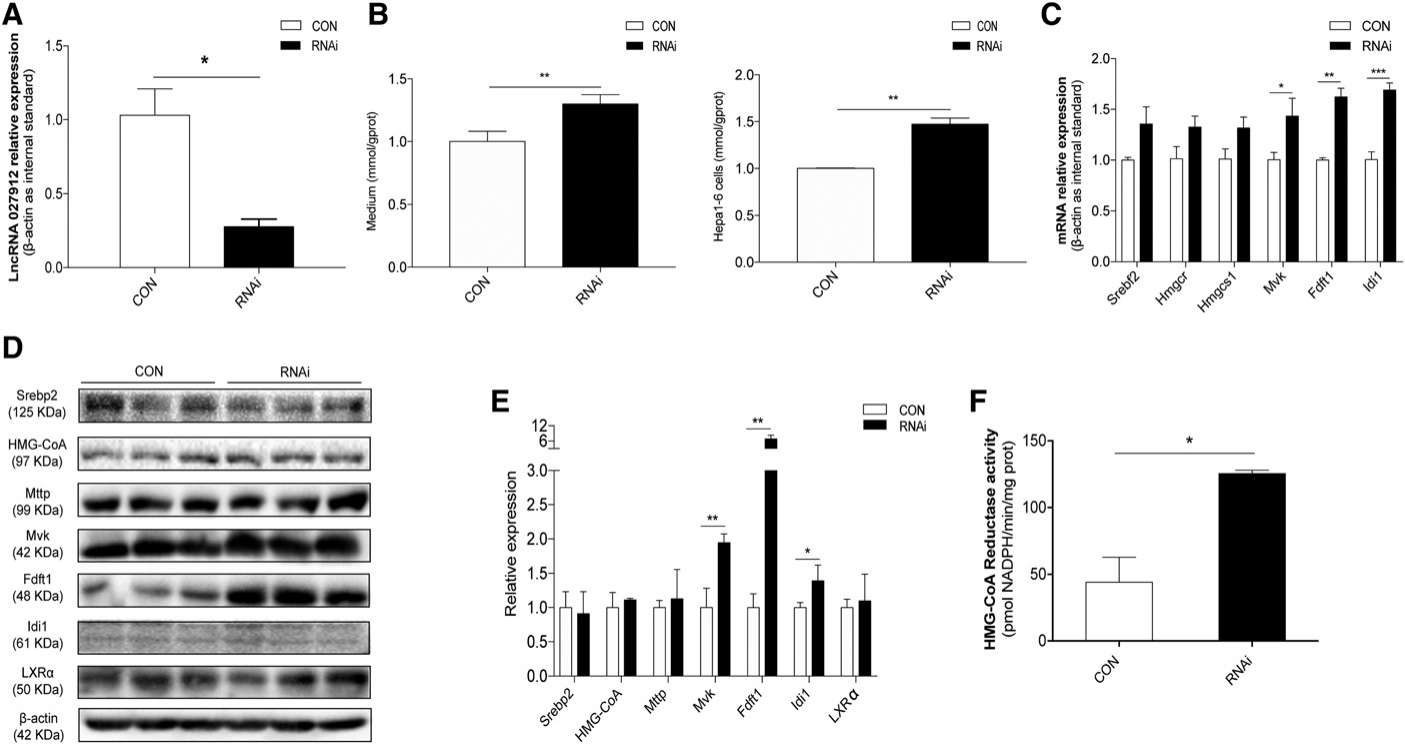
Effect of NONMMUG027912 on hepatic lipid metabolism. A: NONMMUG027912 was efficiently knocked down by 70% in Hepa1-6 cell lines using siRNA oligonucleotides (10 μmol/l) (n = 3, *P < 0.05). B: Total cellular cholesterol levels were significantly elevated in Hepa1-6 cells and culture medium following the knockdown of NONMMUG027912 (n = 3, **P < 0.01). C: Expression of cholesterol biosynthesis-related genes *Mvk*, *Fdft1*, and *Idi1* was significantly increased via the knockdown of NONMMUG027912 in Hepa1-6 cells (n = 3, *P < 0.05, **P < 0.01, ***P < 0.001). D, E: Protein levels of cholesterol synthesis-related proteins Mvk, Fdft1, and Idi1 increased significantly after NONMMUG027912 knockdown (n = 3, *P < 0.05, **P < 0.01). F: HMG-CoA reductase activity significantly increased in the NONMMUG027912 knockdown group as compared with the control group (n = 3, *P < 0.05).

Due to the abundant expression of NONMMUG027912 in Hepa1-6 cells compared with AML12 cells (supplemental Table S6), NONMMUG027912 was knocked down in Hepa1-6 cells using a siRNA oligonucleotide to investigate its effect on cholesterol levels and fatty accumulation. As shown in **Fig. 7A**, NONMMUG027912 expression significantly decreased after knockdown. Efficient knockdown of this lncRNA caused no notable change in fat accumulation or in the transcription levels of the fatty acid synthesis-related genes, *Srebf1*, *Fasn*, *Acly*, or *Acaca* (supplemental Fig. S3). Nevertheless, NONMMUG027912 knockdown significantly increased cholesterol levels in Hepa1-6 cells and in the medium (Fig. 7B), as well as the expression of six genes essential in cholesterol metabolism, especially *Mvk*, *Fdft1*, and *Idi1*, at the transcriptional level (Fig. 7C). Moreover, several cholesterol synthesis-related proteins, including Srebp2, HGM-CoA, MTTP, Mvk, Fdft1, Idi1, and LxRα, were detected through Western blotting. As shown in Fig. 7D and E, the protein levels of Mvk, Fdft1, and Idi1 significantly increased after NONMMUG027912 knockdown. Furthermore, HMG-CoA reductase activity also increased significantly in the NONMMUG027912 knockdown group, as shown in Fig. 7F. These results, taken together, suggest that NONMMUG027912 might be upregulated by PPARα to modulate cellular cholesterol biosynthesis.

## DISCUSSION

NAFLD encompasses a spectrum of liver disorders ranging from hepatic steatosis to nonalcoholic steatohepatitis, which may ultimately lead to cirrhosis (29). Numerous metabolic pathways have been shown to be intertwined in the development of NAFLD, including de novo lipogenesis, β-oxidation, hepatic triglyceride accumulation, inflammatory cytokine release, oxidative stress, mitochondrial dysfunction, and endoplasmic reticulum stress (30). Several studies have profiled lncRNAs in NAFLD in both mice and humans but have failed to further elucidate the functions of the candidate lncRNAs (31–33).

Thus, we used high-throughput RNA-seq combined with lncRNA-mRNA correlation analysis to interpret the aberrantly expressed lncRNAs’ functions in HFD-fed mice. The HFD-fed mouse has been widely used as a model in the study of NAFLD (34–36). Subsequently, we utilized cellular assays to connect the identified lncRNAs to a specific metabolic pathway. Based on the GO and KEGG results for the differentially expressed protein-coding RNAs, several biological processes may have been affected by the HFD, such as lipid biosynthesis, fatty acid metabolism, and oxidation/reduction. Further investigation into the identified lncRNAs and protein-coding genes suggested six pairs of lncRNAs/proteins whose juxtaposition and significant level of coexpression suggest a regulatory functional association. Notably, four of these five protein-coding genes (*G0s2*, *Elovl5*, *Elovl6*, and *Mgll*) are metabolic genes regulated by the nuclear receptor, PPARα (19). These findings suggest that the expression of the lncRNAs, NONMMUG002873, NONMMUG027912, NONMMUG043402, and XLOC_105129, might be regulated by PPARα and be involved in hepatic lipid metabolism in conjunction with their coexpressed protein-coding genes. Assays in liver cells demonstrated that these lncRNAs were upregulated by a PPARα agonist.

Given that these lncRNAs may regulate the transcription of *cis* neighboring genes, we selected one of the five lncRNAs, NONMMUG027912, for further functional investigation. Knockdown of NONMMUG027912 did not significantly change the transcription level of the nearby gene, *Elovl6* (supplemental Fig. S4). This lncRNA may regulate another process in lipid metabolism related to PPARα.

Because de novo lipogenesis and cholesterol biosynthesis are crucial processes for the liver, we measured the effects of NONMMUG027912 knockdown on cellular cholesterol and triglyceride contents as well as the expression of metabolic genes. While NONMMUG027912 knockdown did not significantly affect the triglyceride content, it did significantly increase the cellular cholesterol level as well as the expression of cholesterol biosynthesis-related genes and HMG-CoA reductase activity. PPARα is known to govern several biological processes, such as fatty acid uptake and intracellular binding, mitochondrial β-oxidation, peroxisome fatty acid oxidation, ketogenesis, triglyceride turnover, gluconeogenesis, and bile synthesis/secretion (37). PPARα agonists, such as fibrate, reduce triglyceride levels but not cholesterol levels in the serum of patients with hyperlipidemia (38). Our finding that the lncRNA, NONMMUG027912, was upregulated by a PPARα agonist suggests that this lncRNA may be the mediator connecting PPARα to cholesterol homeostasis.

In this study, we systemically explored the transcription profile of lncRNAs in HFD-fed mice and set up a pipeline to identify functional lncRNAs by integrating genome-wide screening of the lncRNA transcriptome in a pivotal metabolic organ under pathophysiological metabolic conditions. Using this approach, we have identified a lncRNA, NONMMUG027912, that is potentially regulated by PPARα and is involved in the process of cholesterol biosynthesis.

## Supplementary Material

Supplemental Data
