# Supplementary material for: Hepatic transcriptome analysis from HFD-fed mice defines a long noncoding RNA regulating cellular cholesterol levels
Source: J Lipid Res. 2018 Nov 30;60(2):341–52. doi: 10.1194/jlr.M086215 (PMC6358296; doi:10.1194/jlr.M086215)
Supplement: Supplemental Data [file supp_60_2_341__index.html]

Hepatic transcriptome analysis from HFD-fed mice defines a long noncoding RNA regulating cellular cholesterol levels — Supplemental Data 

# Hepatic transcriptome analysis from HFD-fed mice defines a long noncoding RNA regulating cellular cholesterol levels

## Supplemental Data

- Supplemental Figure S3 (.docx, 774 KB) - Characterization of NONMMUG027912 and its effect on hepatic lipid metabolism.
- Supplemental Figure S4. (.docx, 59 KB) - Expression of NONMMUG027912 and Elovl6 in siRNA treated
- Supplement Table S1 (.docx, 16 KB) - Composition of chow and high fat diets fed to mouse
- Supplement Table S2 (.docx, 18 KB) - Primer pairs selected for validation by qRT-PCR
- Supplemental Table S3 (.docx, 18 KB) - Hepatic steatosis score
- Supplemental Table S4 (.docx, 18 KB) - Summary of reads mapping to the mouse genome
- Supplemental Table S5 (.docx, 18 KB) - Statustics of assembling transcripts.
- Supplemental Table S6 (.docx, 17 KB) - Relative expression of lncRNA 027912 in AML12 and Hepa1-6
