# Supplementary material for: Hepatic transcriptome analysis from HFD-fed mice defines a long noncoding RNA regulating cellular cholesterol levels
Source: J Lipid Res. 2018 Nov 30;60(2):341–52. doi: 10.1194/jlr.M086215 (PMC6358296; doi:10.1194/jlr.M086215)
Supplement: Supplemental Data [file 10.1194_M086215_jlr.M086215-1.docx]

**Supplemental Figure S3. Characterization of** **NONMMUG027912 and its effect on hepatic lipid metabolism.**


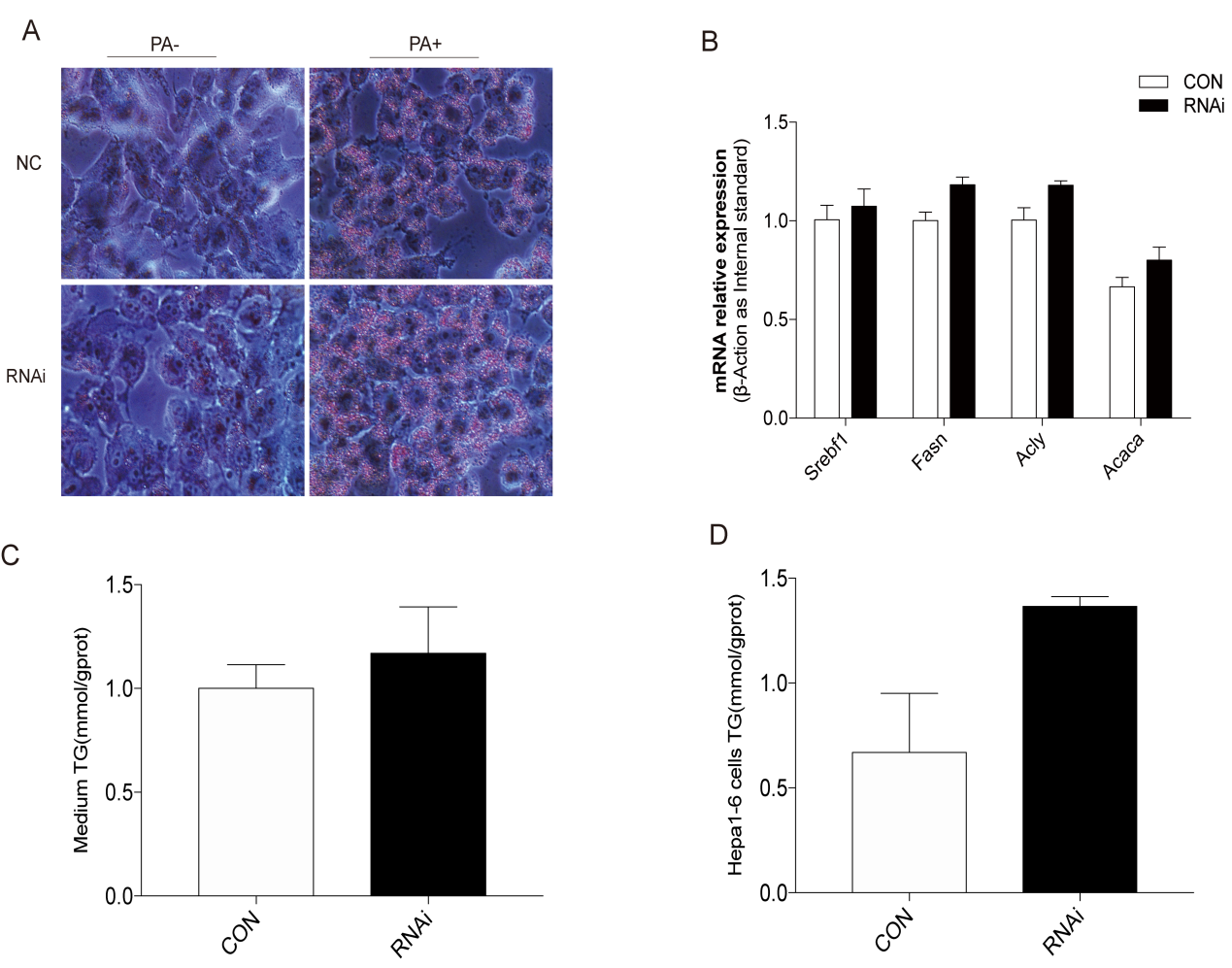


(A) No significant change of lipid accumulation in Hepa1-6 was observed via the knockdown of NONMMUG027912 with or without palmitate acid treatment. (B) Expression of genes associated with lipogenesis was not affected by the knockdown of NONMMUG027912. (C and D) Triglyceride level in medium and Hepa1-6 was not affected by the knockdown of NONMMUG027912.
