# Supplementary material for: Hepatic transcriptome analysis from HFD-fed mice defines a long noncoding RNA regulating cellular cholesterol levels
Source: J Lipid Res. 2018 Nov 30;60(2):341–52. doi: 10.1194/jlr.M086215 (PMC6358296; doi:10.1194/jlr.M086215)
Supplement: Supplemental Data [file 10.1194_M086215_jlr.M086215-2.docx]

**Supplemental Figure S4. Expression of NONMMUG027912 and Elovl6 in siRNA treated**

n=3; *** p<0.001
