# Supplementary material for: Hepatic transcriptome analysis from HFD-fed mice defines a long noncoding RNA regulating cellular cholesterol levels
Source: J Lipid Res. 2018 Nov 30;60(2):341–52. doi: 10.1194/jlr.M086215 (PMC6358296; doi:10.1194/jlr.M086215)
Supplement: Supplemental Data [file 10.1194_M086215_jlr.M086215-3.docx]

**Supplement Table S1. Composition of chow and high fat diets fed to mouse.**

| **Groups** | **Composition (% by kilocalorie)** | **Time** |
| --- | --- | --- |
| **Chow diet** | 70% carbohydrate, 10% fat and 20% protein | 3 months |
| **High fat diet** | 35% carbohydrate, 45% fat and 20% protein | 3 months |
