# Supplementary material for: Hepatic transcriptome analysis from HFD-fed mice defines a long noncoding RNA regulating cellular cholesterol levels
Source: J Lipid Res. 2018 Nov 30;60(2):341–52. doi: 10.1194/jlr.M086215 (PMC6358296; doi:10.1194/jlr.M086215)
Supplement: Supplemental Data [file 10.1194_M086215_jlr.M086215-4.docx]

**Supplement Table S2. Primer pairs selected for validation by qRT-PCR**

| Gene | Forward primer | Reverse primer |
| --- | --- | --- |
| NONMMUG002873 | AGAGCTTGCTCTGCCTCAAC | CGGTCTATCGATGCCAGTGT |
| NONMMUG027912 | GGAAGCAGAGGTAGGTGTAT | GGCTTCCAAGTTCAACAGTC |
| NONMMUG043402 | TTCTCGCTCATGGCCTCTGT | CAGCATCCACCTCCTCTGAA |
| NONMMUG043404 | GGAGGCCTTCTTCTGATGTC | ACTTCTAGGCCATCGGTGGA |
| XLOC_015129 | CTCTCCAACTTCGCAGTGAT | CAGGAGCAGGATTCACATTC |
| Elovl5 | ACTGCTTGTGCTGCTGTTAG | TGCCATAGTTCCTGGAAGAC |
| Elovl6 | ATTCCTGGTCTGAGCGTGAG | GCTTCCATGCAGCTTCAGAG |
| G0s2 | CTCTGGCCAAGGAGATGATG | TGCACACCGTCTCAACTAGG |
| Lamb3 | TTGGCTACACCTCACAGTTC | ATGTGCACCTAACCACTGAC |
| Mgll | GATGCCTGAGGCAAGTTCAC | CGATGAAGGAGGACGAAGAG |
| β-actin | CATCCGTAAAGACCTCTATGCCAAC | ATGGAGCCACCGATCCACA |
| Srebf2 | AGGTTAACGAATGGGAGGGG | CTTCAGTCCCAACAACGGTG |
| Hmgcr | CCTGGAGACCCTTGCTGTTA | TCTCCGTGCTGCTTTAAGGA |
| Hmgcs1 | TGCCTTTCCTTTCAGCCAAC | AGCCCCATTCCTTCATCCAA |
| Fdft1 | TGTGCTGCCTCTTGTTTGTC | TGGGTTCCAATCCTGACTCC |
| Mvk | CCACCACACCAACCTAGCTA | ACAGAGGCACCTTACACTCC |
| Idi1 | GGCTGCCATGTGAATGCTAA | GGAGACTAAAGGCAGGCAGA |
| Srebf1 | CCCTGTAGGTCACCGTTTCT | AGCTGGTAAGTGAGGGCATT |
| Fasn | GCTTCGCCAACTCTACCATG | CCATCGCTTCCAGGACAATG |
| Acly | TCTCTGGCTCCTTTCCTTGG | GGAAGCAAAGCGATGAGGAG |
| Acaca | ACCCCTGCTTAGTGTGTACC | CAATGCACGGTCTACTCAGC |
