# Supplementary material for: Hepatic transcriptome analysis from HFD-fed mice defines a long noncoding RNA regulating cellular cholesterol levels
Source: J Lipid Res. 2018 Nov 30;60(2):341–52. doi: 10.1194/jlr.M086215 (PMC6358296; doi:10.1194/jlr.M086215)
Supplement: Supplemental Data [file 10.1194_M086215_jlr.M086215-5.docx]

**Supplemental Table S3. Hepatic steatosis score**

|  | CON1 | CON2 | CON3 | HFD1 | HFD2 | HFD3 |
| --- | --- | --- | --- | --- | --- | --- |
| Steatosis grade | 0 | 0 | 0 | 2 | 2 | 3 |
| Lobular inflammation | 1 | 0 | 0 | 2 | 1 | 0 |
| ballooning | 0 | 0 | 0 | 1 | 2 | 3 |

Refering to NASH Clinical Research Network Scoring System Definitions.

1)Steatosis grade: Low- to medium-power evaluation of parenchymal involvement by steatosis <5% was scored 0; 5%-33% was scored 1;>33%-66% was scored 2;>66% was scored 3.

2)Lobular inflammation: Overall assessment of all inflammatory foci. No foci was scored 0; 2 foci per 200 field was scored 1; 2-4 foci per 200 field was scored 2; 4 foci per 200 field was scored 3.

3)Ballooning: None was scored 0;Few balloon cells were scored 1; Many cells/prominent ballooning were scored 3.
