# Supplementary material for: Hepatic transcriptome analysis from HFD-fed mice defines a long noncoding RNA regulating cellular cholesterol levels
Source: J Lipid Res. 2018 Nov 30;60(2):341–52. doi: 10.1194/jlr.M086215 (PMC6358296; doi:10.1194/jlr.M086215)
Supplement: Supplemental Data [file 10.1194_M086215_jlr.M086215-6.docx]

**Supplemental Table S4. Summary of reads mapping to the mouse genome.**

| Sample ID | Total reads | Total Mapped Reads | Mapped ratio | Total Unmapped Reads | Unmapped ratio |
| --- | --- | --- | --- | --- | --- |
| CON1 | 57535140 | 49594432 | 86.20% | 7940708 | 13.80% |
| CON2 | 54224158 | 47941180 | 88.41% | 6282978 | 11.59% |
| CON3 | 61471180 | 52805738 | 85.90% | 8665442 | 14.10% |
| HFD1 | 47028818 | 37644282 | 80.05% | 9384536 | 19.95% |
| HFD2 | 53678970 | 46587216 | 86.79% | 7091754 | 13.21% |
| HFD3 | 52707158 | 43347993 | 82.24% | 9359165 | 17.76% |
