# Supplementary material for: Hepatic transcriptome analysis from HFD-fed mice defines a long noncoding RNA regulating cellular cholesterol levels
Source: J Lipid Res. 2018 Nov 30;60(2):341–52. doi: 10.1194/jlr.M086215 (PMC6358296; doi:10.1194/jlr.M086215)
Supplement: Supplemental Data [file 10.1194_M086215_jlr.M086215-7.docx]

**Supplemental Table S5. Statustics of assembling transcripts.**

| Sample ID | Total transcripts | Total genes | Multi exon transcripts | Multi exon transcripts/total transcripts |
| --- | --- | --- | --- | --- |
| CON1 | 31919 | 24983 | 18084 | 56.66% |
| CON2 | 25436 | 19479 | 17202 | 67.63% |
| CON3 | 21782 | 16366 | 17083 | 78.43% |
| HFD1 | 20764 | 15674 | 16294 | 78.47% |
| HFD2 | 22517 | 16858 | 17004 | 75.52% |
| HFD3 | 23281 | 17553 | 16877 | 72.49% |
| Merge | 33803 | 19274 | 25889 | 76.59% |
