# Supplementary material for: Hepatic transcriptome analysis from HFD-fed mice defines a long noncoding RNA regulating cellular cholesterol levels
Source: J Lipid Res. 2018 Nov 30;60(2):341–52. doi: 10.1194/jlr.M086215 (PMC6358296; doi:10.1194/jlr.M086215)
Supplement: Supplemental Data [file 10.1194_M086215_jlr.M086215-8.docx]

**Supplemental Table S6. Relative expression of lncRNA 027912 in AML12 and Hepa1-6**

| Cell llines | Relative expression(Ct Mean) |
| --- | --- |
| AML12 | LncRNA 027912:30.48-31.45 |
|  | β-actin:15.48-15.91 |
| Hepa1-6 | LncRNA 027912:27.53-27.91 |
|  | β-actin:14.70-15.06 |
